# Plastid genomes of the North American *Rhus integrifolia-ovata* complex and phylogenomic implications of inverted repeat structural evolution in *Rhus* L.

**DOI:** 10.7717/peerj.9315

**Published:** 2020-06-16

**Authors:** Craig F. Barrett

**Affiliations:** Department of Biology, West Virginia University, Morgantown, WV, USA

**Keywords:** Sumac, Chloroplast genome, Phylogeny, SNP, Anacardiaceae, Sapindales

## Abstract

Plastid genomes (plastomes) represent rich sources of information for phylogenomics, from higher-level studies to below the species level. The genus *Rhus* (sumac) has received a significant amount of study from phylogenetic and biogeographic perspectives, but genomic studies in this genus are lacking. *Rhus integrifolia* and *R. ovata* are two shrubby species of high ecological importance in the southwestern USA and Mexico, where they occupy coastal scrub and chaparral habitats. They hybridize frequently, representing a fascinating system in which to investigate the opposing effects of hybridization and divergent selection, yet are poorly characterized from a genomic perspective. In this study, complete plastid genomes were sequenced for one accession of *R. integrifolia* and one each of *R. ovata* from California and Arizona. Sequence variation among these three accessions was characterized, and PCR primers potentially useful in phylogeographic studies were designed. Phylogenomic analyses were conducted based on a robustly supported phylogenetic framework based on 52 complete plastomes across the order Sapindales. Repeat content, rather than the size of the inverted repeat, had a stronger relative association with total plastome length across Sapindales when analyzed with phylogenetic least squares regression. Variation at the inverted repeat boundary within *Rhus* was striking, resulting in major shifts and independent gene losses. Specifically, *rps19* was lost independently in the *R. integrifolia-ovata* complex and in *R. chinensis*, with a further loss of *rps22* and a major contraction of the inverted repeat in two accessions of the latter. *Rhus* represents a promising novel system to study plastome structural variation of photosynthetic angiosperms at and below the species level.

## Introduction

The plastid genomes (plastomes) of green plants have been used as phylogenetic tools and studied from an evolutionary standpoint for decades (Soltis, Soltis & Doyle, 1992; Chase et al., 1993; Moore et al., 2007; Ruhfel et al., 2014; Gitzendanner et al., 2018; Li et al., 2019a). Across angiosperms, these genomes are thought to be highly conserved in structure, with two single copy regions (the large single copy, or LSC; and small single copy region, or SSC) and a large inverted repeat with two copies (IRb and IRa) (Palmer & Stein, 1986; Shinozaki et al., 1986; Sugiura, 1992). High throughput sequencing technologies have spurred an explosion of plastid genome sequencing, which is changing the view that these genomes are evolutionarily static, instead revealing the dynamic nature of their evolution (Wicke et al., 2011; Chaw & Jansen, 2018). This increase in plastome sequencing allows investigation of evolutionary dynamics at increasingly finer-scale taxonomic levels, for example, at or below the species level (Barrett, Wicke & Sass, 2018).

The angiosperm order Sapindales Juss. ex Bercht. & J. Presl is composed of nine families and approximately 6,570 species, including many important crops (e.g., citrus, cashew, mango) and numerous ecologically and economically important trees and shrubs. Within the family Anacardiaceae R. Br. (with ~700 accepted species) the genus *Rhus* L. (sumacs, with ~35 species) has received significant attention in phylogenetic and biogeographic studies (Miller, Young & Wen, 2001; Yi, Miller & Wen, 2004, 2007; Andrés-Hernández & Terrazas, 2009; Andrés-Hernández et al., 2014). Despite these previous studies, *Rhus* are poorly studied from a genomic perspective. To date, plastid genomes are available for only two representatives of *Rhus*: *R. chinensis* Mill., and *R. typhina* L. (Lee et al., 2016; Kim et al., 2017; Zuo et al., 2019). Interestingly, plastomes of the Asian *R. chinensis* range in size from 149,011–158,809 bp, while the North American *R. typhina* has a plastome of 160,204 bp, revealing striking variation in plastome size within *Rhus*. This genus, though largely uninvestigated from a genomic perspective, holds the potential as a powerful comparative model for the investigation of plastid genome evolutionary dynamics at and below the species level.

*Rhus integrifolia* (Nutt.) Benth. & Hook. f. ex W.H. Brewer & S. Watson and *R. ovata* S. Watson are two shrubby tree species native to the southwestern USA, Baja California, and outlying islands (Barkley, 1937; Young, 1972, 1974). These two species are ecologically important components of coastal scrub and chaparral habitats, respectively. Both form ligno-tuberous root crowns as an adaptation to frequent fires in their native habitat (Sawyer, Keeler-Wolf & Evens, 2009; Montalvo, Riordan & Beyers, 2017). Further, both serve as important ecological resources, providing habitat for wildlife and protecting against erosion (Horton, 1949). The fruits of both species were used as food and a source of tea for indigenous peoples (hence the names “lemonadeberry” for *R. integrifolia* and “sugarbush” for *R. ovata*) (Timbrook, 2007). Further, both are of great horticultural interest as native ornamental shrubs and pollinator resources and are high-value structural entities used in ecological restoration projects (Montalvo, Riordan & Beyers, 2017).

**Figure 1:**
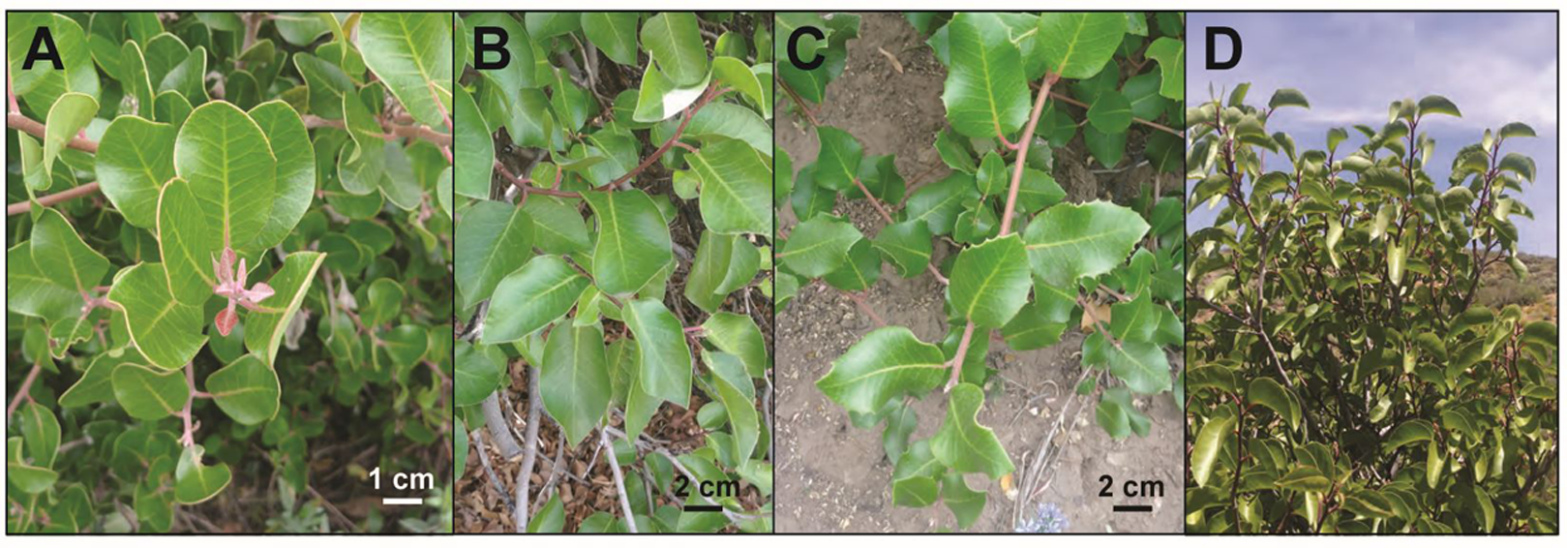
Leaf exemplars of the *R. integrifolia-ovata* complex. (A) *Rhus integrifolia* (Los Angeles County, California, USA). (B) *Rhus ovata* (Gila County, Arizona, USA). (C) Putative hybrid-introgressant between *R. integrifolia* and *R. ovata* showing intermediate leaf morphology. Note the flat, wavy leaf folding and toothed leaf margins. (D) Shrubby habit of *Rhus ovata* (Gila County, Arizona, USA). Photo credits: Craig F. Barrett.

Within *Rhus, R. integrifolia* and *R. ovata* are sister species (Miller, Young & Wen, 2001; Yi, Miller & Wen, 2007) and are known to hybridize frequently in sympatry (Fig. 1), especially in areas of mountainous shoreline where lower-montane chaparral and low-lying coastal scrub habitats abruptly meet (Young, 1974). Both are gynodioecious, yielding hermaphroditic and male-sterile individuals (Young, 1972), yet they widely introgress as evidenced by intermediate morphologies in sympatry. Furthermore, populations of *R. ovata* from central Arizonan and marine Californian chaparral habitats are separated from each other by the Sonoran and Mojave Deserts, which sets up a disjunct distribution of the species (Young, 1974). The extent and frequency of hybrid introgression, patterns of genomic and morphological variation, and ecological niche space of these two species and their hybrid introgressants remain poorly studied despite their ecological and anthropogenic importance. Thus, there is need for future phylogeographic study in this complex, especially in the context of the disjunct distribution of *R. ovata*.

In this study, complete plastid genomes for three accessions from the *R. integrifolia-ovata* complex were sequenced and annotated in order to generate genomic resources for phylogeographic study and to quantify the degree of plastid genomic variation among them. Further, a phylogenomic dataset was constructed and analyzed, consisting of 52 complete, annotated plastid genomes of order Sapindales, emphasizing the family Anacardiaceae and genus *Rhus*. Analysis of these plastomes revealed the extent of sequence variation within the *R. integrifolia-ovata* complex, support for phylogenetic relationships among major clades of Sapindales, dynamic structural changes at the IR-LSC boundary, and independent instances of gene loss within the genus *Rhus*.

## Materials and Methods

### Sampling, DNA extraction, Illumina sequencing, and read quality control

Voucher specimens were collected for one individual of *Rhus integrifolia* (accession CFB 320c CA), and two individuals of *R. ovata* (accessions 290b CA and 371a AZ; Table 1). Permission to collect samples was provided by California State Parks (DRP065), and the USDA Forest Service (FS-2400-8). Because *R. integrifolia* and *R. ovata* are known to introgress frequently in California, individuals were chosen from localities showing no visible evidence of intermediate morphologies. DNA was extracted from leaf material using a CTAB extraction protocol (Doyle & Doyle, 1987), modified to 1/5 volume. Total genomic DNA was visualized on a 1% agarose gel stained with ApexSafe loading Dye (Genesee Scientific, San Diego, CA, USA), and quality/quantity of total DNA was verified via NanoDrop spectrophotometry (ThermoFisher, Waltham, MA, USA) and Qubit fluorometry (dsDNA Broad Range Assay, ThermoFisher, Waltham, MA, USA). Library preparation (Nextera DNA Flex Library Prep Kit) and Illumina sequencing on a NextSeq500 were conducted at Global Biologics, LLC (Columbia, MO, USA). Accessions were pooled at equimolar concentrations and sequenced with nine samples from another project. Paired-end reads (2 × 150 bp) were trimmed from 3′ and 5′ ends via a 3-bp sliding window specifying a minimum PHRED score of 20, and Illumina adapters were removed with Trimmomatic v.0.32 (Bolger, Lohse & Usadel, 2014). After processing, a total of 21,723,472 paired-end reads remained for *R. ovata* accession 371a AZ, 10,979,554 reads remained for *R. ovata* accession 320c CA, and 19,342,934 reads remained for *R. integrifolia* accession 320c CA (Table 1). FASTQC (http://www.bioinformatics.babraham.ac.uk/projects/fastqc/) was used to assess read quality before and after trimming and filtering.

**Table 1:** Newly sequenced accessions of *Rhus integrifolia* and *Rhus ovata*.

Species	Acc #	# Reads	L (bp), x-cov	Locality	Lat, Lon, Elev (m)
*Rhus ovata*	290b	21,723,472	160,262, 1,266.5×	Banner Grade, San Diego County, California, USA	33.084277, −116.563568, 306.9
*R. ovata*	371a	10,979,554	160,173, 2,490.9×	Prescott National Forest, Yavapai County, Arizona, USA	34.432816, −112.553975, 1679.7
*R. integrifolia*	320c	19,342,934	160,141, 6,063.9×	El Capitan State Beach, Santa Barbara County, California, USA	33.743931, −118.411408, 46.9

**Note:**

Acc#, accession number for voucher specimens deposited at the West Virginia University Herbarium (WVA); # reads, the number of paired-end Illumina reads remaining after processing; L (bp), length of the complete plastome in base-pairs; x-cov, coverage depth of the plastome; Lat/Lon/Elev (ft), GPS latitude, longitude, and elevation of collection localities in feet above sea level.

### Plastome assembly and annotation

Plastomes were assembled into complete circular chromosomes with the Perl script ‘NOVOPlasty v.3.8’ (Dierckxsens, Mardulyn & Smits, 2016) specifying the following parameters: genome size range = 100,000–200,000 bp; kmer length = 55; insert size = 350 bp; insert range = 2.0; and using *Rhus ovata* plastid *rbcL* (GenBank MF963245) as a starting seed. Initial assemblies revealed coverage depth estimates > 1,000×, so each read pool was down-sampled to approximately 200× using a custom UNIX command before assembly. Total read pools were mapped back to assembled circular chromosomes under stringent parameters in Geneious v.10 (http://www.geneious.com/), allowing no gaps and no mismatches, in order to check the accuracy of assemblies and calculate coverage depth. The inverted repeat boundaries were verified via continuous paired-end read coverage and the self-dotplot function in Geneious. FASTA files of each chromosome were then rearranged into the same syntenic orientation, with the Large Single Copy (LSC), Inverted Repeat B (IRb), Small Single Copy (SSC), and Inverted Repeat A (IRa), respectively.

Complete, finished plastomes were annotated with the Perl script “PGA” (Qu et al., 2019), which uses BLAST+ (Camacho et al., 2009) and a set of previously annotated plastomes (here, *Anacardium occidentale* L. GenBank # KY635877; *Mangifera indica* L. KX871231; *Pistacia weinmaniifolia* J. Poiss. ex Franch. MF630953; *Spondias mombin* L. KY828469; *Toxicodendron vernicifluum* (Stokes) F.A. Barkley MK419151; *Rhus chinensis* MF351625 and *Citrus aurantiifolia* (Christm.) Swingle KJ865401). Annotations were examined in Geneious to identify mis-annotated regions or problems with predicted translations, and then were adjusted in the NCBI Sequin software (https://www.ncbi.nlm.nih.gov/Sequin). Annotated plastomes were submitted to NCBI GenBank under accession numbers MT024991–MT024993 (File S1).

### Plastome alignment, sequence variation, and repeat content

The three complete plastomes were then aligned via the progressiveMAUVE (Darling, Mau & Perna, 2010) plugin for Geneious (using MAFFT v.7 as an alignment software; Katoh & Standley, 2013), relaxing the assumption of collinearity, with gap opening penalty = 3.0 and offset value = 0.5, in order to check for genomic rearrangements. Dispersed repeat content was calculated with REPuter (Kurtz, 2001) via the Bielefeld University Bioinformatics Service (https://bibiserv.cebitec.uni-bielefeld.de/), quantifying the numbers of forward-forward, forward-compliment, forward-reverse, and palindromic repeats with motif lengths >20 bp. A Hamming distance of 3 and e-value cutoff of 10^−3^ were specified during the search. Perfect tandem repeat content for motifs from 2 to 50 bp were quantified with the Phobos plugin for Geneious 10 (*Mayer C.*, Phobos 3.3.11, 2006–2010, http://www.rub.de/ecoevo/cm/cm_phobos.htm). Information content per locus (here termed “IC”) for each spacer, intron, or protein-coding region yielding variation was calculated as: IC = ((# SNP + # Indels)/locus length) × 100. These were ranked by locus length, and length vs. IC were plotted for each locus to identify highly variable regions within the typical target range for PCR amplification and Sanger sequencing with a single primer pair (e.g., 1,000–500 bp). Primers were designed using the Primer3 (Untergasser et al., 2012) plugin for Geneious. Additional information on sequence variation among *Rhus* accessions was calculated in DnaSP v.6. (Rozas et al., 2017).

### Plastid phylogenomic analysis

A representative sample of species was chosen from complete, annotated plastid genomes of Sapindales to investigate plastid genomic structural variation across the order. This included 52 completely sequenced and annotated plastomes (49 from NCBI GenBank plus the three newly generated *Rhus* plastomes), and two outgroup taxa: *Bretschneidera sinensis* Hemsl. GenBank # NC_037753 (Brassicales Bromhead, Akaniaceae Stapf) and *Shorea pachyphylla* Ridl. ex Symington MK841940 (Malvales Juss., Dipterocarpaceae Blume). In order to make the analyses computationally tractable, a single member of each available genus was included representing seven of the nine families of Sapindales (Biebersteiniaceae and Kirkiaceae had no publicly available complete plastomes). Multiple species of some genera were included due to notable plastome length variation, including: *Commiphora* Jacq. (Burseraceae Kunth); *Rhus* (Anacardiaceae); *Acer* L. and *Dipteronia* Oliv. (Sapindaceae Juss.); and *Entandrophragma* C. DC. (Meliaceae Juss.). IR annotations were added to plastomes missing this information in GENEIOUS via the self-dotplot function, and the second IR copy was removed from all accessions (IRa). The fifty-four complete plastomes (minus IRa) were aligned with progressiveMAUVE as above. The number of syntenic, locally collinear blocks (LCB) was calculated and subjected to a pairwise “double cut and join” (DCJ) analysis in MAUVE to estimate and visualize the number of genomic rearrangements and major inversions.

In order to interpret plastome evolution in a comparative context, a phylogenetic tree was constructed for a concatenated matrix of the seven largest LCB identified in MAUVE (ranging from 11,663 to 52,267 bp aligned length) for a total of 209,579 bp in aligned length, including both coding and non-coding regions. RAxML (Stamatakis, 2014) was used to generate a tree under the GTR-Γ model, with the default number of rate categories (25), and base frequencies estimated empirically. Ten replicate tree searches were conducted, starting from parsimony trees, to check for convergence in topology. Then, 1,000 standard bootstrap replicates were conducted in RAxML under the same search parameters and displayed on the maximum likelihood tree estimate. All analyses were run across 30 cores, each using of 16 GB RAM (specifying RAxMLHPC-PTHREADS) on a Thinkmate VSX R5 760V3 server-class workstation (Thinkmate, Waltham, MA, USA).

To investigate the relationship between and total plastome length, IR length, and total tandem repeat content, all accessions were compared via phylogenetic least-squares regression (PGLS), which accounts for phylogenetic signal in trait covariance among species (Felsenstein, 1985; Garland, Harvey & Ives, 1992; Grafen, 1992), using the R packages “ape” (Paradis & Schliep, 2019), “phytools” (Revell, 2012), and “caper” (Orme et al., 2013). Tandem repeat content for all 54 complete plastomes (excluding IRa) was calculated in TandemRepeatsFinder (Benson, 1999) specifying a minimum alignment score of 50, and alignment parameters of 2, 7, and 7 for matches, mismatches, and indels, respectively. The consensus size of each repeat (bp) was multiplied by the number of each repeat to calculate the total tandem repeat content in bp for each plastome. Phylogenetic signal for IR length, repeat content, and total plastome length was calculated as Pagel’s λ and its significance compared to a model of λ = 0 (Pagel, 1999). PGLS analysis was run under a Brownian Motion model, with λ estimated. Lastly, a closer investigation of expansion and contraction of IR boundaries was conducted using IRscope (Amiryousefi, Hyvönen & Poczai, 2018), an R ‘Shiny’ application that allows analysis of up to ten annotated plastomes simultaneously (https://irscope.shinyapps.io/irapp/), and outputs a JPEG illustration of IR boundary dynamics.

## Results

### Plastome structure, repeat content, and sequence variation

Assembled plastome sequences for *Rhus* ranged from 160,141 bp (*R. integrifolia*) to 160,262 bp (*R. ovata* from CA) (Table 1; File S1). All three plastomes contained a total of 110 genes: 76 protein coding genes (CDS), 30 transfer RNA genes, and four ribosomal RNA genes. The LSC region ranged from 87,980 bp in *R. integrifolia* to 88,086 in Arizonan *R. ovata*; the IR from 26,602 in *R. integrifolia* to 26,635 bp in Californian *R. ovata*; and the SSC from 18,880 bp in Arizonan *R. ovata* to 18,957 bp in *R. integrifolia*. There was a total of 111 segregating sites (excluding sites with gaps), and an average pairwise nucleotide diversity (π) of 0.00055 ± 0.00016. The number of nucleotide substitutions in pairwise comparisons ranged from 127 between Californian and Arizonan *R. ovata* to 208 between Californian *R. ovata* and *R. integrifolia* (Table 2). All three accessions had similar numbers of dispersed and tandem repeats (Table 3; File S2). Forward–Forward dispersed repeats were the most common, followed by palindromic, forward–reverse, and forward–compliment based on our search criteria. Tandem repeats were abundant, with pentanucleotide repeats being most common, while dinucleotide repeats were the rarest, considering repeat motifs from 2 to 20 bp (Table 3). Of particular note was a 22 bp minisatellite repeat within the *ndhC-trnV*^*UAC*^ spacer that varied in copy number between the three *Rhus* accessions (ATT TTT TTT ATT ATT AAT TAT T), with one unit in *R. integrifolia*, two units in the Arizonan accession of *R. ovata*, and three in the Californian accession of *R. ovata*.

**Table 2:** The number of pairwise nucleotide differences among three *Rhus* plastomes, excluding Inverted Repeat A.

	*R. ovata* CA	*R. ovata* AZ
*R. ovata* CA		
*R. ovata* AZ	127	
*R. integrifolia*	208	196

**Table 3:** Numbers of dispersed and tandem repeats in three *Rhus* plastomes.

	*R. integrifola*	*R. ovata* CA	*R. ovata* AZ
Dispersed repeats			
Forward–Compliment	1	1	2
Forward–Forward	32	33	33
Forward–Reverse	13	15	16
Palindromic	19	18	18
Total	65	67	69
Tandem repeat motif length (bp)			
2	5	5	5
3	16	17	16
4	76	77	77
5	253	257	256
6	154	157	158
7	47	45	45
8	21	21	21
9	11	11	10
>9	30	31	28
Total	613	621	616

Overall plastome structure and synteny did not differ among all three newly sequenced *Rhus* accessions (File S1). Interestingly, the plastomes of two accessions of *Rhus chinensis* (GenBank accessions MF351625 and KX447140) displayed a major contraction of the IR relative to *R. integrifolia, R. ovata*, *R. typhina*, and another accession of *R. chinensis* (MG267385), with the IR being some 10 KB smaller in the former *R. chinensis* accessions. In the two accessions of *R. chinensis* with a reduced IR, four genes normally found in the IR are instead found in the LSC region: *rpl2*, *rpl23*, *trnI*^*CAU*^, and *ycf2*.

**Figure 2:**
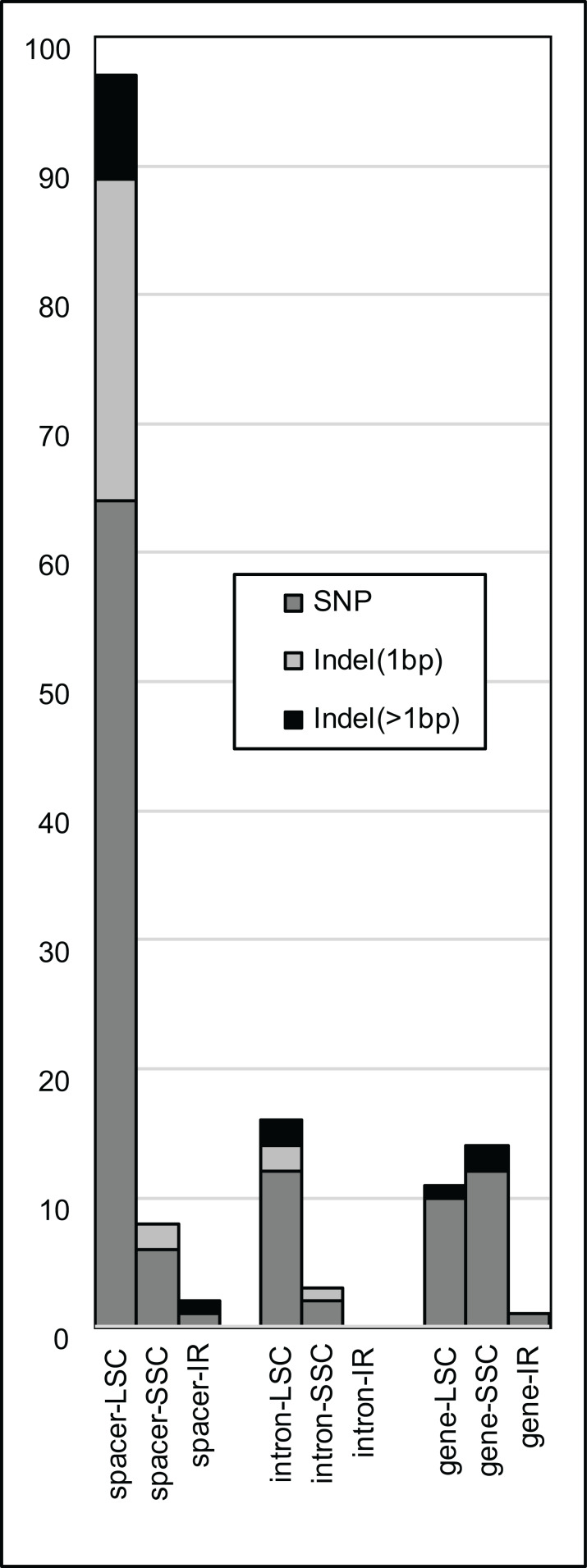
Plastome variation by region. Numbers of single nucleotide polymorphisms (SNPs, dark gray), single-base insertion/deletions (light gray), and insertions/deletions > 1 bp (black) across coding gene regions (i.e., exons, RNA genes), intergenic spacers, and introns of all three aligned plastomes of *Rhus integrifolia* and *R. ovata*.

**Figure 3:**
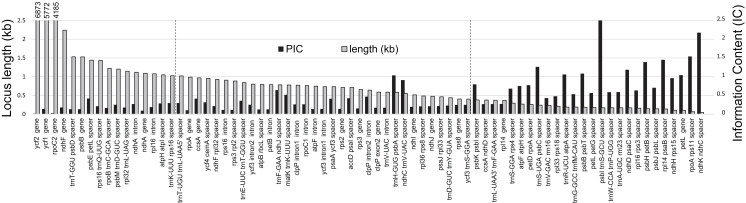
Informative characters by plastid locus. Comparison of informative character content (IC) for all loci containing variation among three *Rhus* accessions. Here, the black columns indicate IC = ((# SNP + # Indels)/locus length) × 100. Gray columns = length of each locus (kilobases; numbers above first three loci are lengths in bp). Vertical dashed lines represent the range for a typical PCR amplicon for Sanger sequencing with two primers (1,000–500 bp).

The majority of SNPs, single-base indels, and multi-base indels occur in intergenic spacers within *R. integrifolia* and *R. ovata*, and these are predominantly found in the LSC region (Fig. 2). SNPs and indels within introns are present mainly in the LSC, followed by the SSC, with none in the IR. Exonic SNPs and multi-base indels are slightly more prevalent in the SSC than in the LSC. Considering information content (IC), several regions display relatively high variation within the range of commonly used PCR and sequencing capabilities with two primers for a single amplicon (i.e., 500–1,000 bp; Fig. 3). These include: the *trnF*^*GAA*^*-ndhJ* spacer, *matK-trnK*^*UUU*^ spacer, *accD-psaI* spacer, *clpP* intron #2, *trnH*^*GUG*^*-psbA* spacer, *psaA-ycf3* spacer, *trnE*^*UUC*^*-trnT*^*GGU*^ spacer, and *ndhC*^*UAC*^*-trnV* spacer. Oligonucleotides for amplification of these eight regions are listed in File S3.

### Plastid phylogeonomics of *Rhus* and order Sapindales

**Figure 4:**
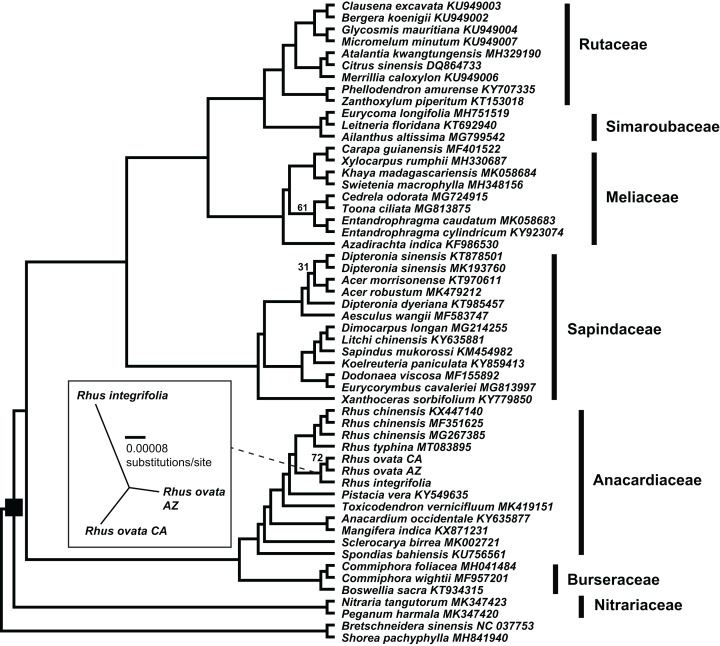
Plastid phylogenetic tree of Sapindales. Maximum likelihood tree of a 209,753 bp alignment of seven concatenated locally collinear plastome blocks across 52 representatives of order Sapindales, representatives from seven of nine constituent families. Black square indicates order Sapindales relative to the two outgroup taxa from Brassicales and Malvales. Values adjacent to branches indicate RAxML bootstrap support; all are 100% unless otherwise noted. Inset: Unrooted Maximum likelihood tree displaying branch length differences based on a whole-plastome alignment of the three *Rhus* accessions sequenced in this study, excluding IRa. Dashed line shows the position of the *Rhus integrifolia-ovata* complex in the larger tree. Scalebar represents substitutions per site under a GTR+Γ model in RAxML.

The concatenated matrix of 54 taxa and 209,573 aligned bp had 26,989 variable positions, excluding gaps, with 16,421 parsimony informative sites, 10,568 singleton sites, and 87,070 sites including at least one inferred gap (File S4). Fig. 4 shows the estimated phylogenetic relationships among the 52 complete plastomes of Sapindales, plus two non-sapindalean outgroups. The Maximum likelihood analysis recovered 100% bootstrap support for all “deep” relationships among families within Sapindales (excluding Biebersteiniaceae Schnitzlein and Kirkiaceae Takhtajan, for which no complete plastomes were available). Rutaceae Juss. were placed as sister to Simaroubaceae DC., followed by Meliaceae. This clade was placed as sister to Sapindaceae, and collectively these four families were placed as sister to a clade of Anacardiaceae + Burseraceae. Nitrariaceae Lindl. grouped as sister to all other families of Sapindales included in this analysis. Within Anacardiaceae, three accessions of the Asian *Rhus chinensis* grouped as sister to an accession of the North American *R. typhina* L. The two accessions of *Rhus ovata* (CA and AZ) were weakly supported as sister to one another (BS = 72), and together sister to *R. integrifolia*. The *Rhus integrifolia-ovata* clade grouped as sister to all other *Rhus* accessions. *Rhus* was grouped as sister to *Pistacia, Toxicodendron, Anacardium* L. + *Mangifera* L., *Sclerocarya* Hochst., and *Spondias* L., respectively.

**Figure 5:**
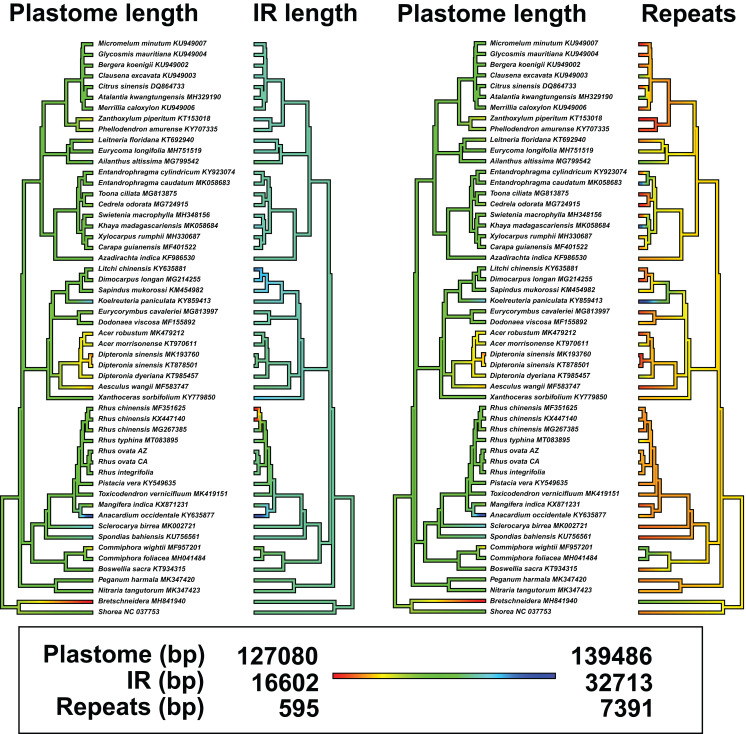
Ancestral state reconstructions of total plastome length, length of the inverted repeat (IR), and tandem repeat content. Analyses were conducted under a Brownian Motion model of trait covariance in the R packages “ape” and “phytools”. Red-orange values in the legend indicate smaller values, while blue values represent larger values.

There was evidence for phylogenetic signal in total plastome length (Pagel’s λ = 0.802, *p* = 0.0017), while there was no signal in IR length (Pagel’s λ = 0.017, *p* = 0.8885) or repeat content (Pagel’s λ = 6.68 × 10^−5^, *p* = 1.0) based on analysis of 54 plastomes. Phylogenetic least squares regression revealed no association between IR length and total plastome length (adjusted *R*^2^ = 0.0079, *F* = 1.421, *p* = 0.2387), but a significant, albeit weak association between repeat content and total plastome length (adjusted *R*^2^ = 0.2175, *F* = 15.73, *p* = 0.0002). A model including both IR length and repeat content as explanatory variables yielded similar results (adjusted *R*^2^ = 0.2256, *F* = 9.722, *p* = 0.0006), wherein repeat content was significantly associated with total plastome length (*p* = 0.0002) and IR length was not (*p* = 0.2189). Many members of Sapindaceae display relatively smaller total plastome lengths (e.g., *Acer, Dipteronia, Aesculus* L.; Fig. 5), but this is not reflected by smaller IR lengths for those species. The plastome of *Anacardium occidentale* was the longest in the dataset, and had the longest IR region, but otherwise there were no clear patterns of variation in IR length driving total length of the plastome.

Synteny analysis in MAUVE of the 54 complete plastomes revealed a total of 11 locally collinear, syntenic blocks (LCB; File S5). There were a few notable genomic rearrangements among members of Sapindales, but these were mostly specific to individual species or genera and were not phylogenetically informative at “higher” taxonomic levels, for example, at the family level or above (File S5). *Mangifera indica* has a large inversion in the LSC region, and *Anacardium occidentale* contains a ~6,700 insertion of mitochondrial DNA within the IR, as observed previously (Rabah et al., 2017). The other striking example of plastome modification comes from two of three accessions of the Asian species *Rhus chinensis*: GenBank accessions KX447140 and MF351625 have IR lengths of 16,741 bp and 16,602 bp, respectively, relative to accession MG267385, which has an IR length of 27,375 bp (Fig. 5; File S5).

**Figure 6:**
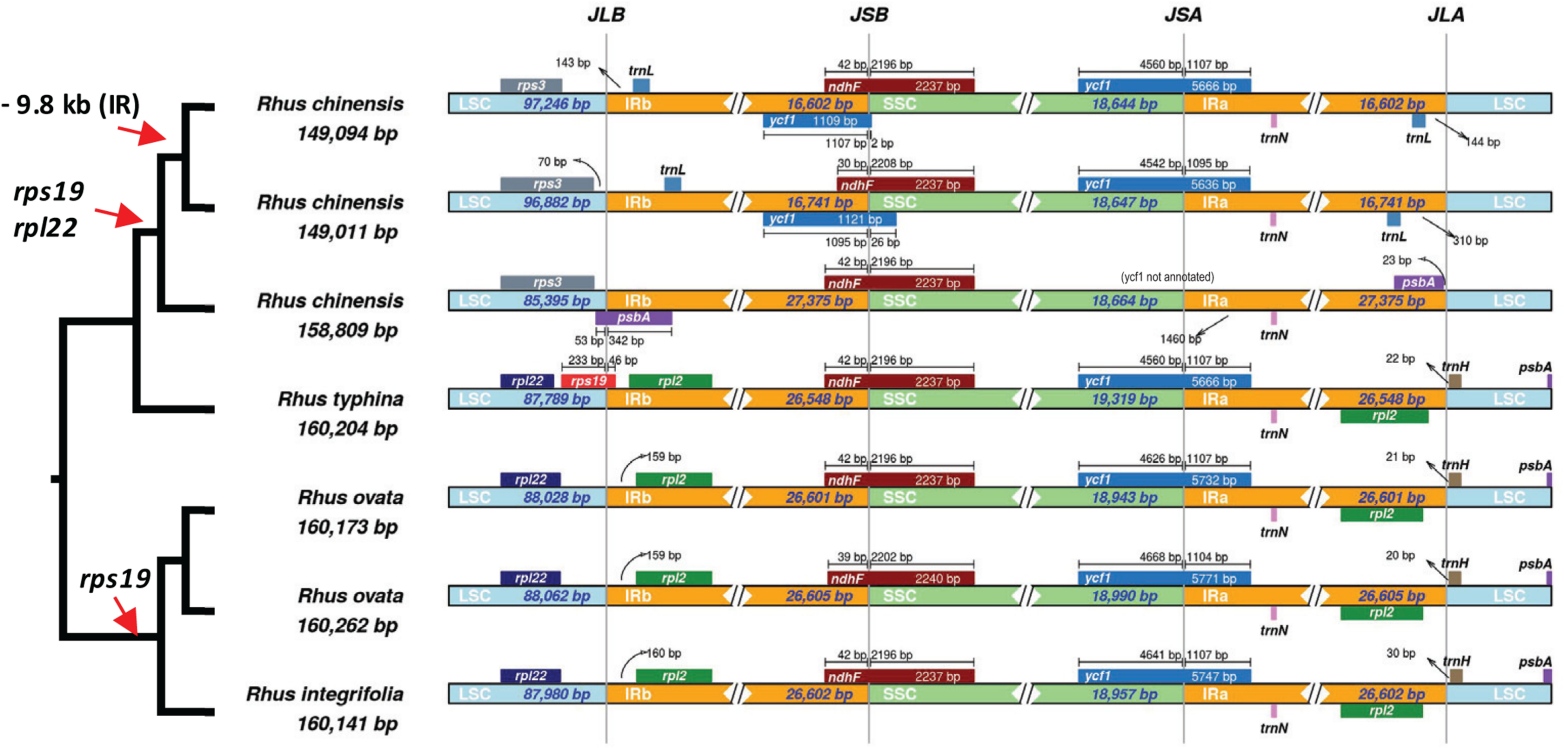
Inverted Repeat (IR) boundaries in *Rhus*. The tree figure (left) corresponds to relationships from Fig. 4, which are all supported with bootstrap values of 100. Red arrows indicate losses of genes or regions of the plastome. LSC, Large Single Copy region; IRb, Inverted Repeat copy “b”; ‘SSC, Small Single Copy Region; IRa, Inverted Repeat copy “a”; JLB, LSC-IRb junction; LSB, IRb-SSC junction; JSA, SSC-IRa junction; JLA, IRa-LSC junction. Small bars and arrows indicate the distance to the nearest IR junction, and diamonds/double-slashes indicate truncations for the purpose of visualization.

A closer analysis of IR boundaries within *Rhus* reveals dynamic expansions and contractions of the IR, primarily at the IR-LSC junctions (Fig. 6). The most notable finding is the independent loss of the *rps19* gene from the plastomes of *R. chinensis* and the *R. integrifolia-ovata* complex, with further loss of *rpl22* in *R. chinensis* (Fig. 6). The IR is expanded at the LSC-IR boundary in one accession of *R. chinensis* relative to *R. integrifolia, R. ovata*, and *R. typhina*, to include a portion of *psbA*, which is typically found at the start of the LSC region (Fig. 6). This is followed by an approximately 9.8 KB contraction of the IR boundary in two accessions of *R. chinensis*. The *rps19* gene spans the LSC-IRb boundary in *Rhus typhina*, while in *R. ovata* and *R. integrifolia*, the gene is missing. Based on the phylogenetic pattern of (*R.integrifolia-ovata*, (*R. typhina*, *R. chinensis*)), it can be inferred that *rps19* was lost at least twice within *Rhus*, based on the current sampling.

## Discussion

Three complete plastid genomes of the *Rhus integrifolia-R. ovata* species complex were sequenced, annotated, and analyzed in a comparative context with several other complete plastomes from the order Sapindales. Patterns of nucleotide polymorphism and indel variation were characterized among the three newly generated *Rhus* plastomes, providing genomic resources for population-level and phylogeographic study of these ecologically and economically important species. Additionally, phylogenetic analysis of 52 complete plastomes in order Sapindales provides resolution and robust branch support among most families within the order, allowing a well-supported basis for phylogenomic comparison of plastid genome evolution. The most striking finding was the dynamic evolution of the inverted repeat boundary in the genus *Rhus*, which was evident based on limited sampling of only seven accessions representing four species of this genus. This included independent losses of the *rps19* gene in *R. ovata-R. integrifolia* complex and in *R. chinensis*, with an additional 9.8 KB contraction of the IR within two of three individuals of *R. chinensis*. These findings suggest that the genus *Rhus* may have experienced several such events, providing a potentially novel, dynamic model system in which to study plastome structural changes in angiosperms at and below the species level.

### Plastome structure, repeat content, and sequence variation in *R. integrifolia* and *R. ovata*

Plastome structure and synteny in the three newly sequenced *Rhus* accessions are somewhat typical for angiosperms (Wicke et al., 2011; Ruhlman & Jansen, 2014). The degree of overall plastome sequence variation among the three *Rhus* accessions is similar to what is observed in other intraspecific studies of plastid genomic variation among photosynthetic angiosperms, for example, in: *Silene vulgaris* (Moench) Garcke (Krüger et al., 2019), *Macadamia integrifolia* Maiden & Betche (Nock et al., 2019), *Quercus acutissima* Carruth. (Zhang et al., 2020), *Utricularia amethystina* Salzm. ex A. St.-Hil. & Girard (Silva et al., 2019), *Capsicum annuum* L. (Magdy et al., 2019), *Holcoglossum* Schltr. spp. (Li et al., 2019b). Repetitive DNA is common in the three *Rhus accessions*, with 65–69 dispersed repeats >20 bp in length) and 613–621 tandem repeats of motif length 2–20 bp (File S2). These regions are candidates for primer design and the study of length variation in a population-genetic or phylogeographic context. In particular, a 22 bp minisatellite repeat within the *ndhC-trnV*^*UAC*^ spacer varied between all three accessions and holds potential as an informative marker. The vast majority of SNP and indel variation is found in the LSC region, particularly within spacer or intron regions. Some of these intron and spacer regions correspond to those shown in previous studies to contain relatively high levels of variation (e.g., *ndhC-trnV*^*UAC*^; Shaw et al., 2005, 2007), and are likely to be useful both above and below the species level as variable markers. *Rhus*-specific rimers for PCR amplification were designed for eight of these regions (File S3). Furthermore, the complete plastid genome sequences for *R. integrifolia* and *R. ovata* can be used to design specific target-capture or long-range PCR probes (Cronn et al., 2012; Mariac et al., 2014; Peñalba et al., 2014; Bethune et al., 2019).

### Plastid phylogeonomics of *Rhus* and order Sapindales

Several previous studies have addressed phylogenetic relationships among families of the order Sapindales, or have included representatives of Sapindales in larger studies of angiosperm relationships (Muellner, Vassiliades & Renner, 2007; Weeks et al., 2014; Magallón et al., 2015; Muellner-Riehl et al., 2016; Li et al. 2019b). However, these studies differ in the relative placement of some of the nine families of Sapindales, with many lacking support for key relationships due to the reliance upon either limited genomic information (e.g., few-gene analyses), or by limited taxon sampling. Order Sapindales contains nine families, 479 genera, and approximately 6,570 species, or approximately 2.15% of angiosperm species diversity (Angiosperm Phylogeny Group, 2016; The Plant List, 2010). While the sampling of 52 plastomes in the current study does not capture the species richness of this diverse order, it does provide support for higher-level relationships based on the plastomes included, and further enables a robust framework for comparative plastid genomics in the order. In fact, the relationships among sapindalean families recovered in the current study are corroborated by another recent study based on complete plastome sequences in Wang et al. (2020), here with the addition of two plastomes from Nitrariaceae (*Nitraria* L. and *Peganum* L.), which were not included in the aforementioned study.

Missing from the current study are representatives of two sapindalean families, Biebersteiniaceae and Kirkiaceae. Muellner-Riehl et al. (2016) sampled 207 species representing all families of Sapindales, and conducted a phylogenetic analysis based on three plastid loci: *rbcL*, *atpB*, and the *trnL-trnF* spacer. While most of the deep branch support values in that study were moderate to high, the placement of Biebersteiniaceae and Nitrariaceae among other members of Sapindales received no support. Additionally, there was moderate support for Meliaceae as sister to Simaroubaceae, whereas in the current study and in that of Wang et al. (2020), both based on complete plastome data, Meliaceae is supported as sister to Simaroubaceae + Rutaceae. The topology from the current study among families is also identical to that in (Li et al., 2019b) based on coding regions of the plastome. However, in that study Biebersteiniaceae (not included in the current study) are sister to Nitrariaceae with <50% bootstrap support, collectively sister to the rest of Sapindales. Thus, future phylogenomic studies of Sapindales should focus on dense, strategic plastome sampling representative of the species richness of this clade, as well as on broad representation of nuclear genomic information (Collins, Gostel & Weeks, 2016; Eiserhardt et al., 2018; Johnson et al., 2019; Dodsworth et al., 2019).

Boundary shifts of the IR in plastid genomes are hypothesized to be one of the primary drivers of overall plastid genome size variation (Wicke et al., 2011; Ruhlman & Jansen, 2014). However, this hypothesis was not supported across 52 representative plastomes of Sapindales in a comparative framework via phylogenetic least squares regression. While a few shifts in plastome size were visibly associated with expansions or contractions of the IR (e.g., *Anacardium occidentale, Rhus chinensis*; Fig. 5), shifts in the IR boundary alone are not sufficient to explain overall plastome size variation in Sapindales among phylogenetically independent lineages, based on the current sampling. Variation in repeat content appears to be a stronger determinant of plastome length variation in Sapindales (e.g., as in *Asarum*; Sinn et al., 2018).

Perhaps the most striking finding in the current study is the dynamic nature of evolution of the IR boundary in *Rhus*, based on comparison of complete plastomes across the order Sapindales.

The inverted repeat (IR) region of the plastome is the most conserved in terms of sequence variation (Palmer & Thompson, 1982) relative to the LSC and SSC. However, numerous studies have demonstrated the dynamic nature of boundary shifts in the IR, which often has implications for the evolution of gene content, structural stability, and substitution rates (Zhu et al., 2016). By the inclusion of only seven available accessions of four species in *Rhus*, it is evident that IR boundaries are variable at and below the species levels (Fig. 6). Thus, increased representation of plastomes in this genus and related genera will likely reveal additional variation in plastome structure, making *Rhus* a promising system in which to test hypotheses associated with IR expansion/contraction and the overall structural/substitutional dynamics of the plastome in a phylogenetic, comparative context.

IR boundary shifts often lead to gene duplication, loss, and large-scale syntenic rearrangement, all of which have implications for substitution rates of the genes captured or “released” from the IR (Zhu et al., 2016). In some cases, genes can be lost from the plastome as a result of IR boundary shifts (Wicke et al., 2014; Downie & Jansen, 2015), as is the case in *R. integrifolia-ovata* (*rps19*) and *R. chinensis* (*rpl22* and *rps19*; Fig. 6). It is possible that one or both of these genes were transferred to another genomic compartment, either to the mitochondrial genome or the nuclear genome, where they may still be expressed (Martin & Herrmann, 1998; Selosse, Albert & Godelle, 2001). Alternatively, they may have been lost from the organism altogether, with little to no fitness implications for plastid function. Numerous examples of the loss of such “housekeeping” genes are evident in mycoheterotrophic or parasitic plants which contain plastids that presumably retain function at some level (Wicke et al., 2011, 2013, 2016).

The IR is hypothesized to play a major role in the structural stability of the plastome (Palmer & Thompson, 1982). The IR has been lost several times independently among plant lineages, for example in: conifers (Raubeson & Jansen, 1992; Wu et al., 2011; Wu & Chaw, 2014), legumes (Palmer, Osorio & Thompson, 1988; Lavin, Doyle & Palmer, 1990; but also regained de novo, Choi, Jansen & Ruhlman, 2019), parasitic plants (Wicke et al., 2016), mycoheterotrophic plants (Schelkunov et al., 2015; Yuan et al., 2018; Barrett, Sinn & Kennedy, 2019), members of family Geraniaceae Juss. (Guisinger et al., 2011; Blazier et al., 2016), cacti (Sanderson et al., 2015), and a palm (*Tahina spectabilis* J. Dransf. & Rakotoarinivo; Barrett et al., 2016). However, there is mixed evidence that IR loss leads to syntenic instability, including a lack of evidence from *Erodium* L’Hér. (Geraniaceae; Blazier et al., 2016) and weak evidence within the “IR-lacking clade” of papilionoid legumes that plastome rearrangements are prevalent following the loss of the IR (Sabir et al., 2014).

## Conclusion

Three complete plastid genomes were generated for two ecologically important North American shrub species in the genus *Rhus*. Information from these plastomes was used to design PCR primers for amplifying variable regions and regions containing phylogeographically informative repeats in dense population-level samples (e.g., *ndhC-trnV*). Plastome evolution was investigated in a robustly supported phylogenomic framework, revealing striking variation within *Rhus* in terms of dynamics of the LSC-IR boundary, with multiple, independent instances of gene loss. *Rhus* presents a promising novel system in which to study the dynamics of plastome structural variation in photosynthetic angiosperms at or below the species level, and continued representation of plastomes in this genus is likely to reveal additional changes.

## Supplemental Information

10.7717/peerj.9315/supp-1 Supplemental Information 1 NCBI GenBank flat files for three accessions of *Rhus integrifolia-ovata*.

10.7717/peerj.9315/supp-2 Supplemental Information 2 Dispersed repeat content of the three *Rhus* plastomes.

10.7717/peerj.9315/supp-3 Supplemental Information 3 Oligonucleotide primers designed for eight polymorphic regions of the plastome in *Rhus*.

10.7717/peerj.9315/supp-4 Supplemental Information 4 MAFFT alignment of seven concatenated locally collinear blocks among 52 accessions of Sapindales in NEXUS format.

10.7717/peerj.9315/supp-5 Supplemental Information 5 MAUVE alignment of 52 plastomes (excluding IRa) of Sapindales.

## Additional Information and Declarations

### Field Study Permissions

The following information was supplied relating to field study approvals (i.e., approving body and any reference numbers): Permission to collect samples was provided by California State Parks (DRP065), and the USDA Forest Service (FS-2400-8).

### DNA Deposition

The following information was supplied regarding the deposition of DNA sequences: Newly generated, annotated plastid genomes are available at GenBank: MT024991–MT024993.

### Data Availability

The following information was supplied regarding data availability: Complete plastid genomes of Rhus accessions (GenBank format) are available in File S1. Repeat locations are available in File S2. Primer sequences are available in File S3. The plastid coding gene alignment for Sapindales is available in File S4. MAUVE alignment of 54 plastomes is available in File S5.
