# Supplementary figures and images for: Plastid genomes of the North American Rhus integrifolia-ovata complex and phylogenomic implications of inverted repeat structural evolution in Rhus L
Source: PeerJ. 2020 Jun 16;8:e9315. doi: 10.7717/peerj.9315 (PMC7304433; doi:10.7717/peerj.9315)

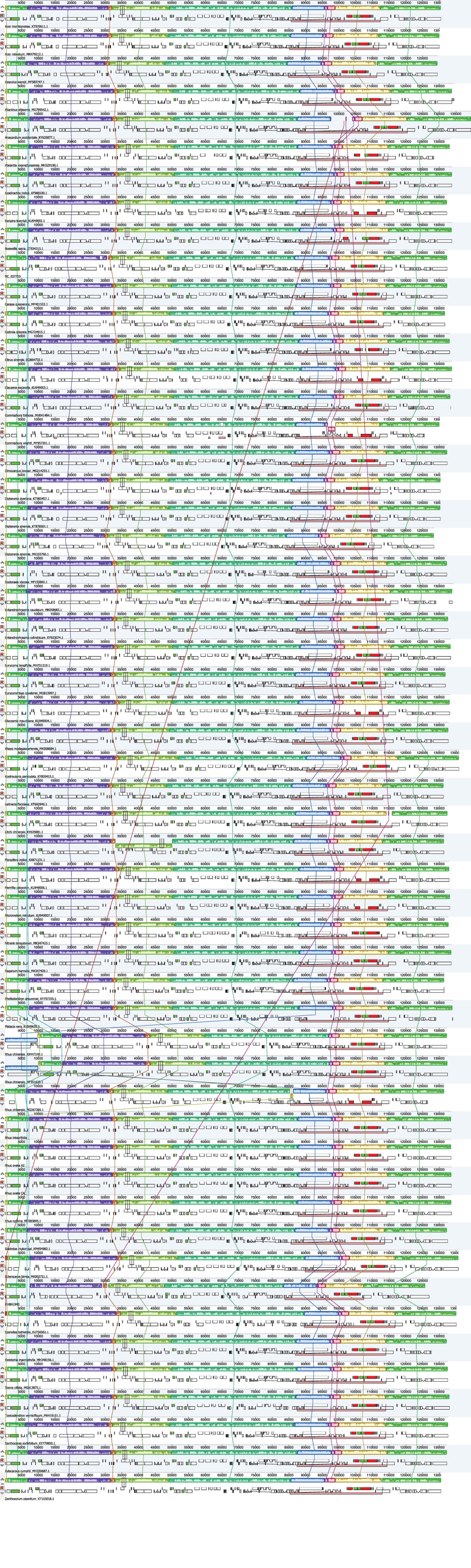

Supplement: Supplemental Information 5 [file peerj-08-9315-s005.jpg]
